# A Multicentric, Single Arm, Prospective, Stratified Clinical Investigation to Confirm MammoWave’s Ability in Breast Lesions Detection

**DOI:** 10.3390/diagnostics13122100

**Published:** 2023-06-17

**Authors:** Daniel Álvarez Sánchez-Bayuela, Navid Ghavami, Cristina Romero Castellano, Alessandra Bigotti, Mario Badia, Lorenzo Papini, Giovanni Raspa, Gianmarco Palomba, Mohammad Ghavami, Riccardo Loretoni, Massimo Calabrese, Alberto Tagliafico, Gianluigi Tiberi

**Affiliations:** 1 Breast Imaging Department, Radiology Service, Complejo Hospitalario Universitario de Toledo, 45007 Toledo, Spain; 2 Faculty of Chemical Science and Technology, Instituto Regional de Investigación Científica Aplicada, University of Castilla—La Mancha, 13001 Ciudad Real, Spain; 3 UBT—Umbria Bioengineering Technologies, 06081 Perugia, Italy; 4 School of Engineering, London South Bank University, London SE1 0AA, UK; 5 AUSL Umbria 2, 06034 Foligno, Italy; 6 IRCCS Ospedale Policlinico San Martino, 16132 Genoa, Italyalberto.tagliafico@unige.it (A.T.); 7 Department of Health Sciences, University of Genoa, 16126 Genoa, Italy

**Keywords:** breast imaging, microwave imaging, breast cancer, dielectric properties

## Abstract

Novel techniques, such as microwave imaging, have been implemented in different prototypes and are under clinical validation, especially for breast cancer detection, due to their harmless technology and possible clinical advantages over conventional imaging techniques. In the prospective study presented in this work, we aim to investigate through a multicentric European clinical trial (ClinicalTrials.gov Identifier NCT05300464) the effectiveness of the MammoWave microwave imaging device, which uses a Huygens-principle-based radar algorithm for image reconstruction and comprises dedicated image analysis software. A detailed clinical protocol has been prepared outlining all aspects of this study, which will involve adult females having a radiologist study output obtained using conventional exams (mammography and/or ultrasound and/or magnetic resonance imaging) within the previous month. A maximum number of 600 volunteers will be recruited at three centres in Italy and Spain, where they will be asked to sign an informed consent form prior to the MammoWave scan. Conductivity weighted microwave images, representing the homogeneity of the tissues’ dielectric properties, will be created for each breast, using a conductivity = 0.3 S/m. Subsequently, several microwave image parameters (features) will be used to quantify the images’ non-homogenous behaviour. A selection of these features is expected to allow for distinction between breasts with lesions (either benign or malignant) and those without radiological findings. For all the selected features, we will use Welch’s *t*-test to verify the statistical significance, using the gold standard output of the radiological study review.

## 1. Introduction

Current breast screening programmes for early cancer detection have been demonstrated to reduce breast cancer mortality; however, there is continuous discussion among the scientific community about several aspects of the current gold standard technique, mammography [1,2,3,4]. It is widely known that mammography (either 2D or recent developments such as digital breast tomosynthesis (DBT)) limits its use in population-based screening programmes to both a very specific age range and a limited screening frequency, usually biennial (the ECIBC’s Guidelines Development Group recently recommended women from 45 to 74 years of age [5] and the United States Preventive Services Taskforce recently drafted recommendations to include screening in women aged between 40 and 75 years of age [6]). Meanwhile, newer studies estimate that breast cancer (BC) is diagnosed in 6.6% of women younger than 40 years old [7], and around 20% of BC cases in Europe occur in women when they are below the age of 50 [8]. Overdiagnosis in screening, the use of ionising X-rays, and the cumulative effect it places on women have led to many controversies and discussions. Moreover, breast density is a big concern among radiologists as tumors may be mammographically hidden in dense breasts [9,10,11] or if the breast comprises small, elongated salt-like microcalcification particles [12]. In addition, the discomfort caused by breast compression and the performance reduction in dense breasts have motivated many researchers to investigate novel, safe techniques that overcome the mentioned limitations [13]. In this regard, microwave imaging has emerged as an interesting potential alternative to ionising-based techniques [14,15], based on the ability to discriminate between healthy tissues and tissues with lesions based on existing contrast in dielectric properties (permittivity and conductivity) within the microwave frequency spectrum.

This paper aims to present the approved clinical protocol for analysing one of these operational microwave systems, named MammoWave (developed by UBT Srl, Rivotorto di Assisi, Italy), which functions in air with only one transmitting and one receiving azimuthally rotating antenna, operating within the frequency band 1–9 GHz [16,17,18]. In contrast to mammography, the microwave exam is performed with the woman lying down on an examination table, in a comfortable prone position, without breast compression. The acquisition time of the system is roughly 7 min per breast.

For instance, this clinical protocol proposes a prospective multicentre international clinical trial to confirm the ability of MammoWave in breast lesions detection; it represents another step for finding suitable clinical applications through novel techniques such as microwave imaging, e.g., it represents a starting point prior to evaluating these kinds of systems in screening environments. Before starting such clinical trials, this detailed clinical protocol outlines all aspects of the study. Here, some of the key aspects of this protocol will be presented and discussed including its primary and secondary objectives and outputs, the inclusion and exclusion criteria, the number of participants to fulfil the required sample size, the statistical approach, the hospitals involved in the study, and the regulatory path followed. An overview of MammoWave’s current clinical validation status is also provided with a detailed description of the device. In more detail, to evaluate MammoWave in the framework of this clinical trial, microwave imaging will be performed on volunteers who have already gone through conventional exams and radiologists’ review to be used as the reference standard. For each breast of each volunteer undergoing this trial, our algorithm based on Huygens’ principle will be used to create a conductivity-weighted microwave image. Next, several microwave image parameters, i.e., features, will be calculated and used to measure and quantify the images’ non-homogenous behaviour. A selection of these features is expected to permit the distinction between breasts with radiological findings (WF) and those without any radiological findings (NF).

## 2. Experimental Design

### 2.1. Clinical Protocol

The sponsored prospective multicentre international clinical trial entitled “Clinical Investigation to Confirm the Ability of MammoWave in Breast Lesions Detection” was initiated (ClinicalTrials.gov Identifier: NCT05300464) with the aim of quantifying the capability of MammoWave in discriminating between breasts with no lesions or radiological findings (NF) and breasts with radiological findings (WF), that may be benign or associated with breast cancer. The protocol was designed following the SPIRIT reporting guidelines [19]. The study was approved by the Ethics Committee of CEAS Umbria (24197/22/AV, 9 March 2020), which acts as a coordinator, as it is the ethics committee to which Ospedale San Giovanni Battista—USL Umbria 2, Foligno, Perugia (Italy) refers. Additionally, the study was approved by the Ethics Committee of CER Liguria (337/2022, DB nr. 12281, 26 September 2022), which is the ethics committee to which Azienda Ospedaliera Universitaria IRCCS San Martino, Genova (Italy) refers. Concerning approval in Spain, the site (Complejo Hospitalario Universitario de Toledo, Servicio de Salud de Castilla—La Mancha) reviewed and accepted the approval of the coordinator.

### 2.2. Study Design

#### 2.2.1. Inclusion and Exclusion Criteria

Participants fulfilling all of the following inclusion criteria are eligible for the study:

1. Signed informed consent form;
2. Women;
3. Adult ≥18 years old;
4. Having a radiologist study output (to be considered as the reference standard) obtained using conventional exams within the last month. The conventional exam may be a breast specialist visit and mammography and/or ultrasound and/or magnetic resonance imaging, which will be integrated with the histological one if deemed necessary by the responsible investigator and when available;
5. Volunteers willing to comply with the study protocol and recommendations;
6. Women with intact breast skin (i.e., without bleeding lesions or scars);

The presence of any one of the following criteria will lead to the exclusion of the participant:

1. Women who are participating in another clinical study;
2. Women who belong to any vulnerable group (e.g., women with disabilities or impairments);
3. Women with implanted electronic devices;
4. Women who have undergone a biopsy less than one week before the MammoWave scan;
5. Women with breast implants;
6. Women with nipple piercings (unless they are removed prior to examination);
7. Participation in other studies in the last month before screening;
8. Pregnancy or breastfeeding.

#### 2.2.2. Primary and Secondary Objectives

**Figure 1:**
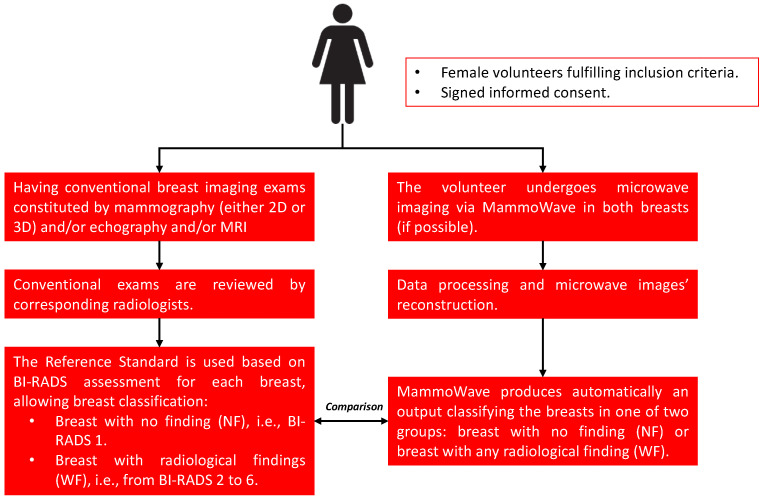
The clinical trial’s workflow. For each breast of each subject, the microwave imaging output of MammoWave will be compared to the output of the radiologist study review (from conventional exams).

The primary objective and expected outcome of this protocol is to generate empirical evidence for the detection of WF breasts (breasts with any kind of lesion, either benign or malignant) by using MammoWave and evaluating its sensitivity (number of ‘true positive’ results) against the reference standard (Figure 1). In addition, MammoWave’s safety and tolerability will be assessed as a safety objective.

The study’s secondary objectives are included in this protocol to further evaluate MammoWave’s performance by assessing: (1) the specificity of MammoWave in NF breast detection against the reference standard, (2) the sensitivity of the MammoWave device in breast lesion detection among different breast densities, (3) the sensitivity of MammoWave in women who had recently undergone a mammographic exam, (4) the sensitivity of MammoWave in breast cancer detection, (5) volunteers’ satisfaction after undergoing the MammoWave exam, and (6) both the specificity and sensitivity of MammoWave (against the reference standard) obtained retrospectively using MammoWave data from RadioSpin [20] technology simulator/artificial intelligence (AI) algorithms.

#### 2.2.3. Number of Participants

The study is composed of two phases: in the first phase, 15 participants in each centre all with NF breasts without any lesions will be examined by MammoWave. These data will be used to calibrate the image parameters’ thresholds for each apparatus installed at each centre. In the second phase, the remaining participants will be enrolled and examined by MammoWave, and the results will be compared against the reference standard collected by the clinical investigator.

According to the sample size calculation, considering that the primary objective of the study is to determine the ability of MammoWave to detect BL, it is anticipated that this study will enroll both patients with BL diagnosis and patients with NF, with a prevalence of BL patients of ~50%; of this 50%, about 50% will be BC patients. The required overall detection ability is 70% (all kinds of breast density). A minimum total number of 350 patients (175 with BL) is required to verify a sensitivity of 70% (H_1_) versus H_0_ = 60%, with an error of first type α = 0.05 and a power (1−β) = 80%. Note that BL includes malignant lesions related to breast cancer (BC) and benign lesions (both may be palpable or non-palpable lesions). BL also includes isolated clustered microcalcifications. Since it has been verified that the detection may depend on breast density and to generally increase the accuracy of the estimation process, the total number of patients to be enrolled has been increased to a maximum of 600. The volunteers’ enrolment started in February 2023 and will presumably last 12 months.

#### 2.2.4. Participating Centres

Three centres (two in Italy and one in Spain) will take part in this study. The two Italian hospitals are: Ospedale San Giovanni Battista—USL Umbria 2, Foligno, Perugia and Azienda Ospedaliera Universitaria IRCCS San Martino, Genova. Complejo Hospitalario Universitario de Toledo (Servicio de Salud de Castilla—La Mancha) will host the clinical study in Spain.

#### 2.2.5. Recruitment and Data Collection

Recruitment will be performed in each centre as deemed appropriate by the clinical investigators in a one-day visit approach (Table 1), including both symptomatic (diagnosed with a lesion, either benign or malignant) and asymptomatic women. The following volunteers’ information will be collected during the clinical study in a dedicated database using a unique identifier for each participant:

- Age;
- Assessed breast (right or left);
- Qualitative breast density according to the Breast Imaging-Reporting and Data System (BI-RADS): A for entirely fatty breasts, B for breasts with scattered areas of fibroglandular density, C for heterogeneous dense breasts, and D for extremely dense breasts [21];
- Radiologists’ conventional study (mammographic and/or echography and/or magnetic resonance imaging) will be collected using BI-RADS notation. This will allow for classifying breasts as NF when the BI-RADS score is 1 and as WF breasts when the BI-RADS score is different from 1, with 2 being for benign lesions, 3 being for follow-up lesions, 4 being for suspicious lesions, 5 being for a highly suspicious lesion, likely to be breast cancer, and 6 being for lesions with confirmed cancer diagnosis [21];
- Histological output will also be collected (when available) using standard classifications;
- MammoWave final output will be collected as NF (breast without relevant findings) or WF (breast with findings);
- Satisfaction questionnaires related to MammoWave use.

#### 2.2.6. Study’s Ethical Conduct

The study will be carried out in accordance with the approved protocol and the principles of the current version of the Declaration of Helsinki, the guidelines of Good Clinical Practice (GCP) issued by the ICH, the European Directive on medical devices 93/42/EEC and ISO Norm 14155 and ISO 14971, the European Law, and Italian and Spanish regulatory authorities’ requirements.

#### 2.2.7. Participants’ Privacy and Confidentiality

The investigators affirm and uphold the principle of the participants’ right to privacy. In particular, the anonymity of the participants will be guaranteed when presenting the data at scientific meetings or publishing them in scientific journals.

## 3. Materials and Equipment

### 3.1. MammoWave Device and Imaging Algorithm

**Figure 2:**
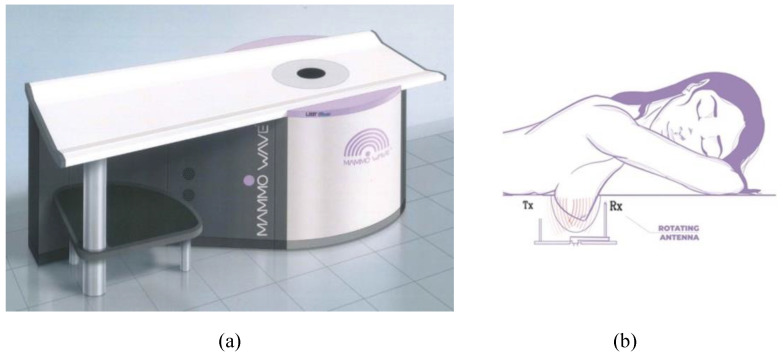
MammoWave system (**a**) and sketch of the breast imaging configuration showing a woman’s breast inside the cup and the transmitting and receiving rotating antennas (**b**).

The first MammoWave prototype was successfully developed and validated on phantoms in 2015. Ever since, MammoWave has undergone several optimization cycles until arriving at the construction of the current clinically ready prototype. The device’s image and configurations are depicted in Figure 2. MammoWave has two antennas, one horn-type antenna acting as a transmitter and the other (Vivaldi-type) acting as a receiver of microwave signals. The two antennas always operate in air (without the need for any matching liquid or medium), have a voltage standing wave ratio <3 in the frequency band of 1–9 GHz, and are vertically positioned at the same height. A 2-port vector network analyser (Copper Mountain, Indianapolis, IN) is used to operate the antennas, both of which are contained in a cylindrical hub surrounded internally using microwave absorbers. The cylindrical hub comprises a cup positioned within a hole, permitting insertion of the woman’s breast while lying in a prone position, as sketched in Figure 2.

To support all patients, three different cup sizes are available, and the best fit for the patient’s breast is selected. The largest cup has a diameter of 135 mm. The cups have the following features. They are made using polylactic acid (PLA) to ensure biocompatibility and with a width of 1 mm. This 1 mm thickness is based on modeling and experimental investigations by the team which demonstrated that this thickness has no effect on the microwave imaging outcomes.

Both antennas rotate around azimuthally, collecting the signals in a multi-bistatic mode. For each transmitting position, the receiving antenna rotates with a 4.5° step, collecting the received signals at 80 positions around the breast. In total, 10 transmitting positions, grouped into 5 doublet sections with centres at 0°, 72°, 144°, 216°, and 288°, are used in the acquisition configuration [17]. MammoWave takes approximately 7 min to complete the acquisition for one breast.

To process the received signals, we use our Huygens-principle-based imaging algorithm [22], which has previously displayed promising preliminary results for several biomedical application areas [17,23,24,25]. This algorithm can reconstruct the images corresponding to a target inclusion inserted in a background medium through only measuring the external surface field of the object under study. The measured field is subsequently back propagated inside the medium (breast) employing the Green’s function to reconstruct the internal field. As the last step, the final intensity image is produced by an incoherent summation of information obtained from all the transmitting positions and frequency points.

### 3.2. Previous Clinical Studies

**Figure 3:**
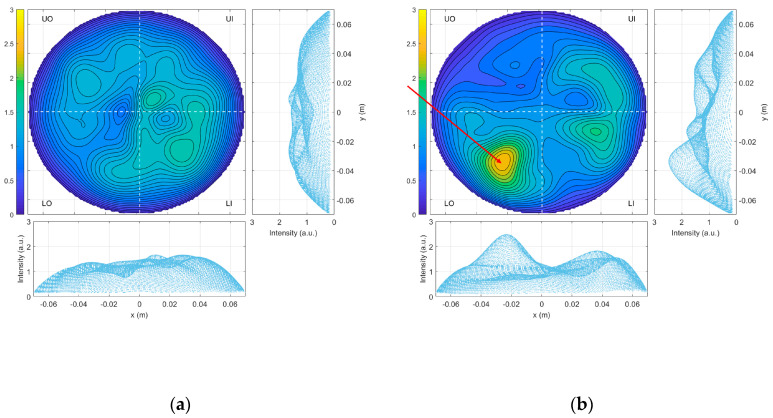
Microwave images (in arbitrary units) using conductivity value 0.3 for: (**a**) an ACR B healthy breast and (**b**) an ACR C breast with a lesion (the red arrow indicates the position of the lesion, confirmed by the conventional radiologist study review to be a 10 mm nodular thickening area).

In a previous prospective clinical trial, a sensitivity of 82.3% and a specificity value of approximately 50% were achieved [17,26]; a retrospective analysis has also been performed, applying supervised machine learning (SML), enhancing specificity to >90% [27]. Figure 3a,b shows examples of obtained microwave images for a breast without any type of lesion and a breast with the described lesion, respectively.

## 4. Detailed Procedure

### 4.1. Microwave Images Analysis

MammoWave acquisition is made just once; then, a set of multiple conductivity-weighted microwave images may be produced, using different values of sigma, and employing rotation subtraction artifact removal algorithms [17,23]. Here, we will first use microwave images produced using sigma = 0.3 S/m and a rotation subtraction artifact removal of 9° degrees; this results in the generation of one microwave image.

Images obtained using the proposed apparatus are intensity maps, given in linear arbitrary units, representing the homogeneity of tissues’ dielectric properties. Images are maximum intensity projection coronal 2D maps of the entire breast volume. To allow for inter and intra-subject comparison, all images are normalised to the unitary average of the intensity.

To allow for quantification of the non-homogenous behaviour of the microwave images, we introduce the following parameters, i.e., features:

MAX = Maximum value of the image;
MIN = Minimum value of the image;
MEA = Mean value of the image;
MED = Median value of the image;
VAR = Variance of the image;
MAD0 = Mean absolute deviation of the image;
MAD1 = Median absolute deviation of the image;
M2AVG = (MAX)/(MEA);
ROS1 = (MAX-MIN)/(MEA-MIN);
ROS2 = (MAX-MIN)/(MED-MIN).

The above-mentioned features are calculated for each conductivity-weighted image on the full image domain denoted with subscript “_i”. Moreover, for each conductivity-weighted image, all listed features except ROS1 and ROS2 are calculated: on the peak region (a region centred in the image’s maximum and extending to MAX/√2), denoted with subscript “_p”, and on its complementary, denoted with subscript “_c”. The ratios between features calculated on the peak region and its complementary are considered as additional features, denoted with subscript “_r”.

Next, following the results of a feasibility study [17], we selected four features: M2MEA_i, MAX_n, VAR_p, MAD0_p, and VAR_r. Thus, for each breast and for all selected features, we introduce a binary score S defined as follows:

$$\begin{array}{c} \mathrm{if}\;\mathrm{feature}>D_{\mathrm{offset}|\mathrm{feature}}\;\mathrm{then}\;S=1\\ \mathrm{if}\;\mathrm{feature}\leq D_{\mathrm{offset}|\mathrm{feature}}\;\mathrm{then}\;S=0 \end{array}$$

The threshold D_offset|feature_ is calculated for each feature, using the median value obtained in each site after recruiting the first 15 volunteers, all with breasts without any lesions. The binary score S is then used for establishing an empirical rule-of-thumb allowing for assessment of the MammoWave images. Specifically, the final assessment of the MammoWave examination is performed as follows:

If the following statement is verified, MammoWave’ final assessment will be “Breast with finding (WF), i.e., with lesion”: (number of S = 1 occurrence) ≥ 5. This MammoWave final assessment is performed prospectively and is blinded with respect to the radiologists’ conventional study outcome.

### 4.2. Statistical Analysis

For all of the selected features of the method given above, we will use the Welch’s *t*-test (i.e., a two-sample two-tailed unpooled variances *t*-test) with α = 0.05 to verify the statistical significance (*p* < 0.05), using the gold standard output of the radiological study review. For all the selected features of the method given above, we will also numerically evaluate the receiver operating characteristic (ROC); the area under the curve (AUC) will be also determined.

The performance of the proposed rule-of-thumb will be evaluated by empirically calculating the true positive rate, i.e., sensitivity (percentual estimates of true WF detection, with a 95% confidence interval), and the true negative rate, i.e., specificity (percentual estimates of true NF detection, with a 95% confidence interval), using the gold standard output of the radiological study review (excluding the first 15 patients of each site). Additional analysis will be performed through stratifying by breast density.

### 4.3. Retrospective Analysis

As stated in the secondary objectives, the specificity and sensitivity of MammoWave will also be measured (against the reference standard) when retrospectively using MammoWave data in one RadioSpin technology simulator/Artificial Intelligence (AI) algorithm. In more detail, RadioSpin (Deep oscillatory neural networks computing and learning through the dynamics of RF neurons interconnected by RF spintronic synapses) is one of the projects funded by the EU within the framework of FUTURE EMERGING TECHNOLOGIES (FET) H2020-FETPROACT; the RadioSpin project aims to build a hardware neural network, as a “Hardware Artificial Intelligence”.

## 5. Expected Results and Discussion

Microwave imaging has emerged as a promising new technology for breast radiology, circumventing patients’ discomfort and the usage of ionising radiation. It employs low-power radio-frequency signals without any breast compression. Due to its safe nature, it has the potential to become very relevant, especially in screening, for increasing coverage of the female population and offering an effective and safe alternative for early breast cancer detection. Specifically, it can be seen as a complementary solution for making screening programmes more inclusive, since microwave imaging may be employed without any safety limitations such as the patient’s age or frequency of use. Its implications and impact could be especially observable in increasing early-stage cancer detection and reducing interval cancers.

MammoWave, the imaging device presented in this work, has recently received its CE mark and ISO certification, enabling it to undergo further clinical research following the “post marked” clinical trials and paving the way for its adoption as a complementary imaging technique. The microwave images produced by MammoWave are intensity maps in linear arbitrary units, which represent the homogeneity of the breast’s dielectric properties. The device avoids the usage of any patient-specific estimation, meaning that it generates the breast images without any a priori knowledge of patient-specific breast dielectric properties, using only the free space dielectric properties in the algorithm.

A possible limitation of this work is that although the persons responsible for clinical trial execution are trained to ensure the correct positioning of the breast inside the cup (i.e., nipple placed in the cup’s centre), the exact breast positioning for the duration of the MammoWave exam cannot be precisely monitored and controlled in this study. Breasts’ shape and volume, which may affect microwave images, are not controlled; the cups are selected by sight. Additionally, this investigation does not consider the subjects’ pre-menstrual information, which may be relevant, especially in young women.

Within this trial, clinical management decisions are based on the results of the radiologists’ conventional study outcome only (following the path for new imaging procedures for breast cancer as suggested in [28]).

Finally, we note that, together with the primary and secondary objectives of the protocol, the key goals of these clinical trials are: (a) to verify if several parameters (features) acquired from the conductivity-weighted microwave images can permit distinction between NF and WF breasts, (b) to verify if an appropriate combination and use of microwave image features may achieve performance enhancement versus single feature, and (c) to retrospectively use the obtained microwave data for testing a novel artificial intelligence hardware system and verify its application for microwave imaging.

This prospective clinical trial on both NF and WF breasts may pave the way for introducing microwave imaging into clinical practice, with the aim of using it for helping in breast lesion identification in asymptomatic women of any age, without any safety restrictions. The outcome of this prospective study will be compared to those obtained using the MARIA device (Micrima, UK) and the Wavelia system (MVG, France) reported in [29,30,31].

## Figures and Tables

**Table 1:** Participants’ timeline, including enrolment and clinical assessments in a single-visit design.

	Visit 1
Signed written informed consent	**X**
Demography	**X**
Medical History	**X**
Inclusion/exclusion criteria	**X**
Qualitative breast density assessment	**X**
Standard breast evaluation	**X**
MammoWave examination	**X**
Satisfaction questionnaire (after MammoWave examination)	**X**
Adverse event (if any)	**X**

## Data Availability

Not applicable.
